# Supplementary material for: Causal Relationship between Aging and Anorexia Nervosa: A White-Matter-Microstructure-Mediated Mendelian Randomization Analysis
Source: Biomedicines. 2024 Aug 16;12(8):1874. doi: 10.3390/biomedicines12081874 (PMC11351342; doi:10.3390/biomedicines12081874)
Supplement: Supplementary file 1 [file biomedicines-12-01874-s001.zip › Supplementary Figure/Figure S9/Leaveoneout plot of MR analysis for ubm-b-1937 to AN.pdf]

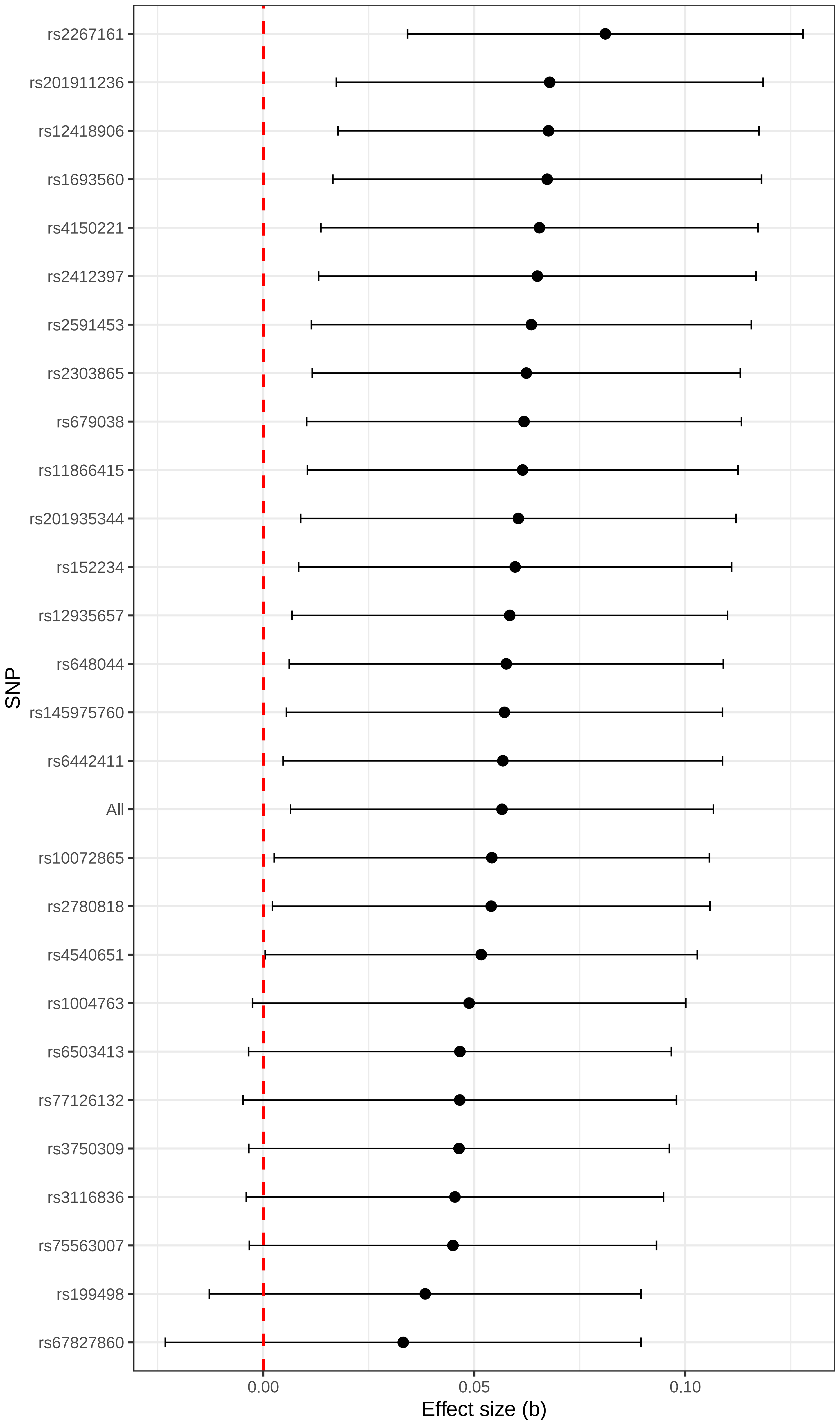

rs2267161
rs201911236
rs12418906
rs1693560
rs4150221
rs2412397
rs2591453
rs2303865
rs679038
rs11866415
rs201935344
rs152234
rs12935657
rs648044
rs145975760
rs6442411
All
rs10072865
rs2780818
rs4540651
rs1004763
rs6503413
rs77126132
rs3750309
rs3116836
rs75563007
rs199498
rs67827860
SNP
0.00
0.05
0.10
Effect size (b)
